# Unravelling the mechanotransduction pathways in Alzheimer’s disease

**DOI:** 10.1186/s13036-023-00336-w

**Published:** 2023-03-28

**Authors:** Francesca Donnaloja, Emma Limonta, Christian Mancosu, Francesco Morandi, Lucia Boeri, Diego Albani, Manuela Teresa Raimondi

**Affiliations:** 1 Politecnico Di Milano, Department of Chemistry, Materials and Chemical Engineering “G. Natta”, Campus Leonardo, Piazza Leonardo da Vinci 32, 20133 Milan, Italy; 2 Department of Neuroscience, Istituto di Ricerche Farmacologiche Mario Negri IRCCS, Milan, Italy

**Keywords:** Nuclear lamina, Alzheimer’s disease, Extracellular matrix, Nuclear-cytoplasmic transport, Tau, Synaptic loss

## Abstract

Alzheimer’s disease (AD) represents one of the most common and debilitating neurodegenerative disorders. By the end of 2040, AD patients might reach 11.2 million in the USA, around 70% higher than 2022, with severe consequences on the society. As now, we still need research to find effective methods to treat AD. Most studies focused on the tau and amyloid hypothesis, but many other factors are likely involved in the pathophysiology of AD. In this review, we summarize scientific evidence dealing with the mechanotransduction players in AD to highlight the most relevant mechano-responsive elements that play a role in AD pathophysiology. We focused on the AD-related role of extracellular matrix (ECM), nuclear lamina, nuclear transport and synaptic activity. The literature supports that ECM alteration causes the lamin A increment in the AD patients, leading to the formation of nuclear blebs and invaginations. Nuclear blebs have consequences on the nuclear pore complexes, impairing nucleo-cytoplasmic transport. This may result in tau hyperphosphorylation and its consequent self-aggregation in tangles, which impairs the neurotransmitters transport. It all exacerbates in synaptic transmission impairment, leading to the characteristic AD patient’s memory loss. Here we related for the first time all the evidence associating the mechanotransduction pathway with neurons. In addition, we highlighted the entire pathway influencing neurodegenerative diseases, paving the way for new research perspectives in the context of AD and related pathologies.

## Introduction

**Fig. 1:**
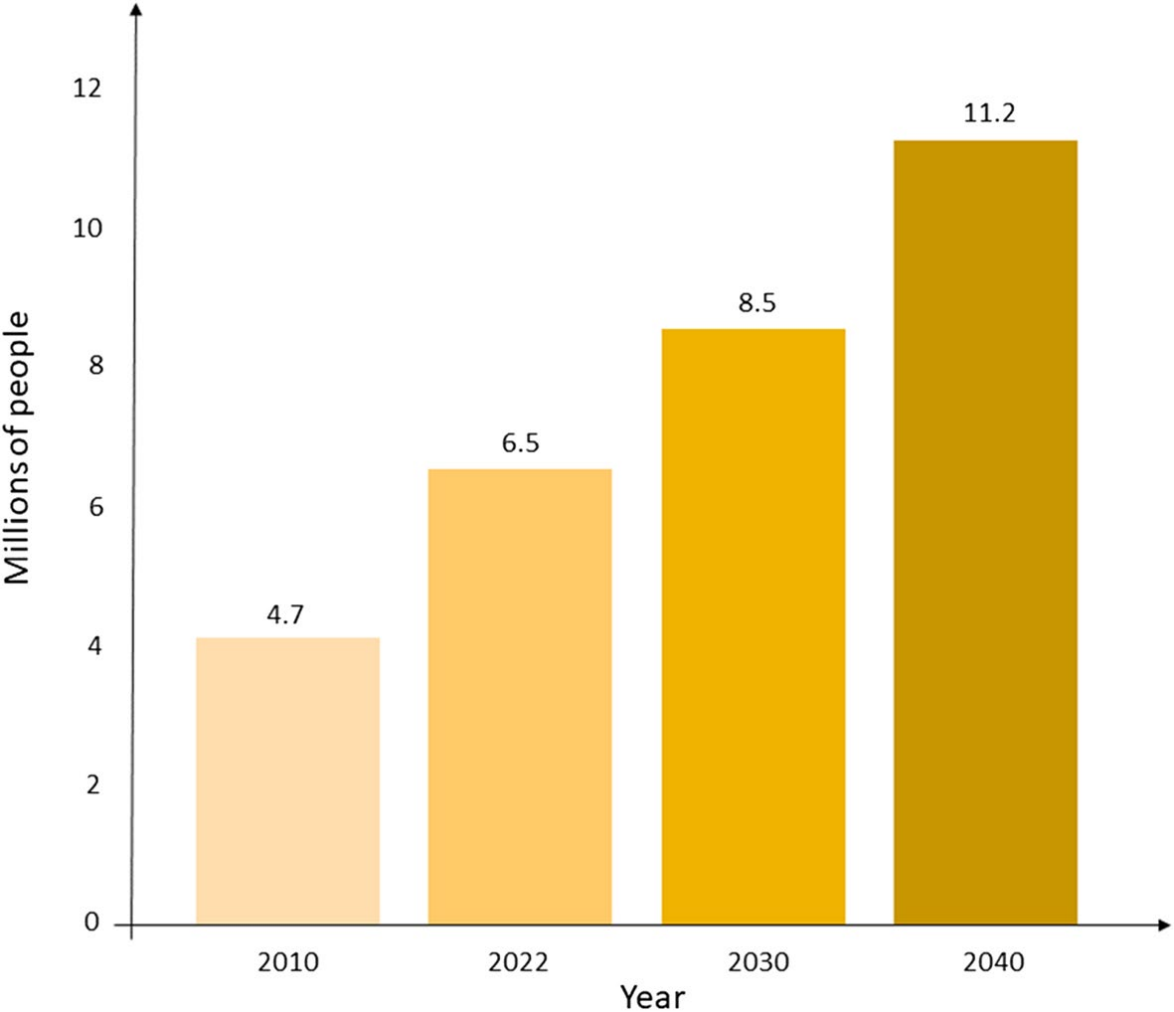
Estimation of U.S. population with Alzheimer’s disease in the next decades. The data are based on a study conducted by the Alzheimer’s Association [1, 8]

**Fig. 2:**
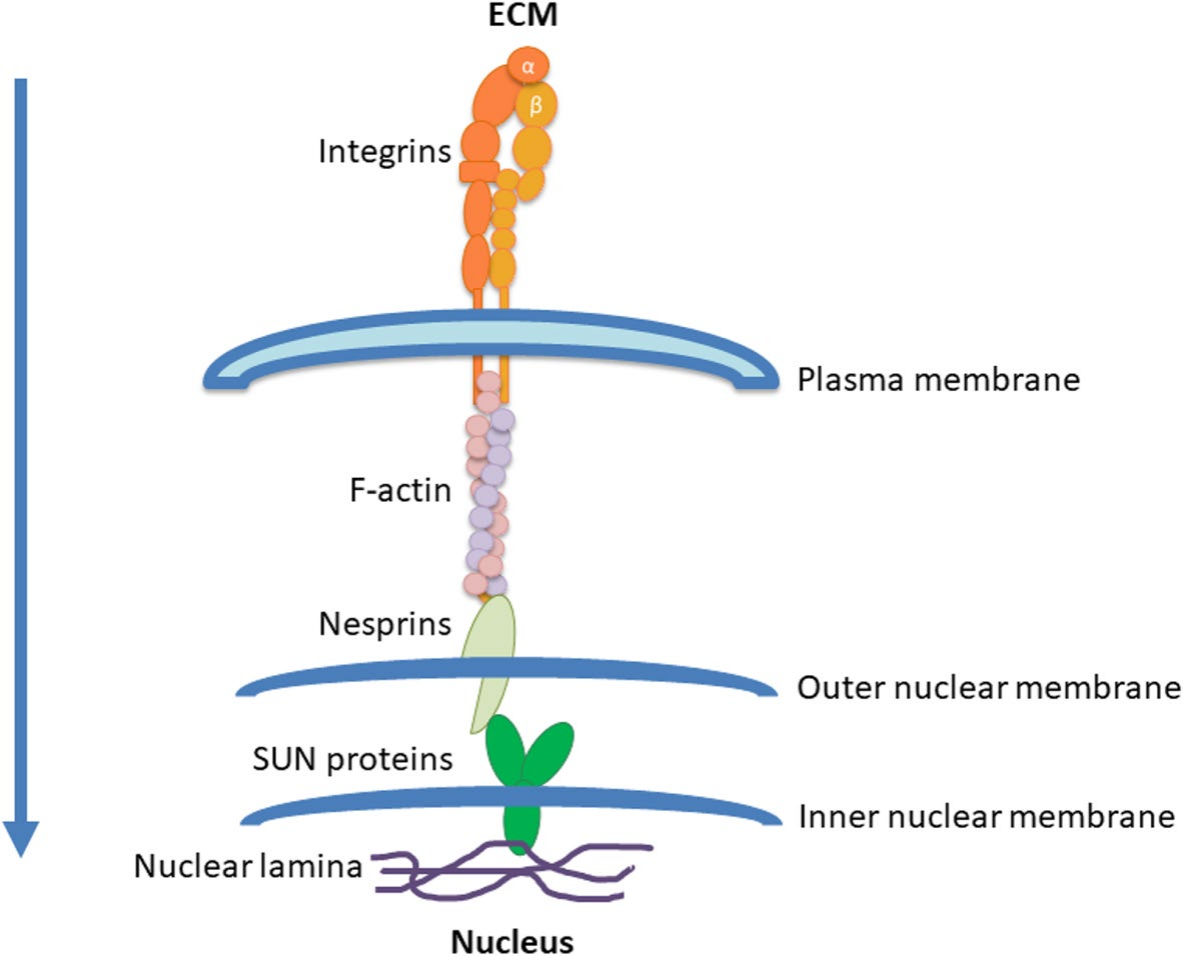
Force transmission pathway from the ECM to the nuclear lamina. Extracellular matrix interacts with integrins that transduce the mechanical stimuli to the cytosolic F-actin through the plasma membrane. The F-actin microfilaments are connected to the nuclear lamina by the LINC complex, consisting of nesprins and SUN proteins. SUN proteins are directly connected to the nuclear lamina

Americans aged 65 and older suffering from Alzheimer’s disease (AD) are estimated to be about 6.5 million [1]. By the end of 2040, U.S. population with dementia might reach 11.2 million of cases, around 70% higher than 2022 (Fig. 1). AD is a neurodegenerative disorder characterized by progressive cognitive impairment with loss of memory and behavioural difficulties [1]. Well-known pathological markers found in AD patients are extracellular aggregates of beta-amyloid (Aβ) and intracellular hyperphosphorylated tau (hyp-tau) deposits. The former lead to the formation of senile plaques, while the latter aggregates are self-organized structures namely tangles, which impair the tau function as stabilizer for microtubules and alter the motor protein-mediated transport [2]. Recently, mechanotransduction has been related to pathological changes occurring during AD progression. Mechanotransduction is the process that converts mechanical stimuli from the extracellular matrix into biochemical signals inside the cell, with consequences on cell structure, gene expression and physiological functions [3]. The extracellular stimuli are propagated into the nucleus via sequential interactions of integrins, F-actins, the Linker of Nucleoskeleton and Cytoskeleton (LINC) complex and the nuclear lamina (Fig. 2) [4–6]. Integrins are heterodimeric transmembrane receptors, which mediate the ECM-cytoskeleton microfilaments connection. The ECM-cytoskeleton link is further strengthened by the presence of other key cytosolic proteins, such as talin and vinculin [7]. The cytoskeleton proximal to the plasma membrane is predominantly composed by F-actin microfilaments and supports the cell structure [7]. F-actin microfilaments are connected to the nuclear lamina by the LINC complex, consisting of nesprins and SUN proteins [5]. Nesprin 1 and nesprin 2 pass through outer nuclear membrane, connecting F-actin to the SUN1, a protein crossing the inner nuclear envelope [4, 5]. Among the SUN protein family, SUN1 was reported to interact with the nuclear lamina [4, 5]. As previously stated, this complex transduction network may have a role in AD.

Even tough specific aspects of mechanotransduction in neurons have already been discussed, to the best of our knowledge, there is no work summarizing the entire pathway from ECM to synapse response. To fill this gap, this review summarizes evidence dealing with this hypothesis and explains how the extracellular matrix affects the nuclear lamina and how this may be associated to the impaired synaptic activity affecting AD neurons. To this end, we firstly analyzed literature highlighting the alteration of single components of the mechanotransduction pathway. In particular, we report the changes in ECM, nuclear lamina, nuclear transport and synaptic activity in AD. We then discuss incisive connections between the single players, defining a hypothetic mechanism of the mechanotransduction-driven disease progression.

### Extracellular matrix (ECM)

**Fig. 3:**
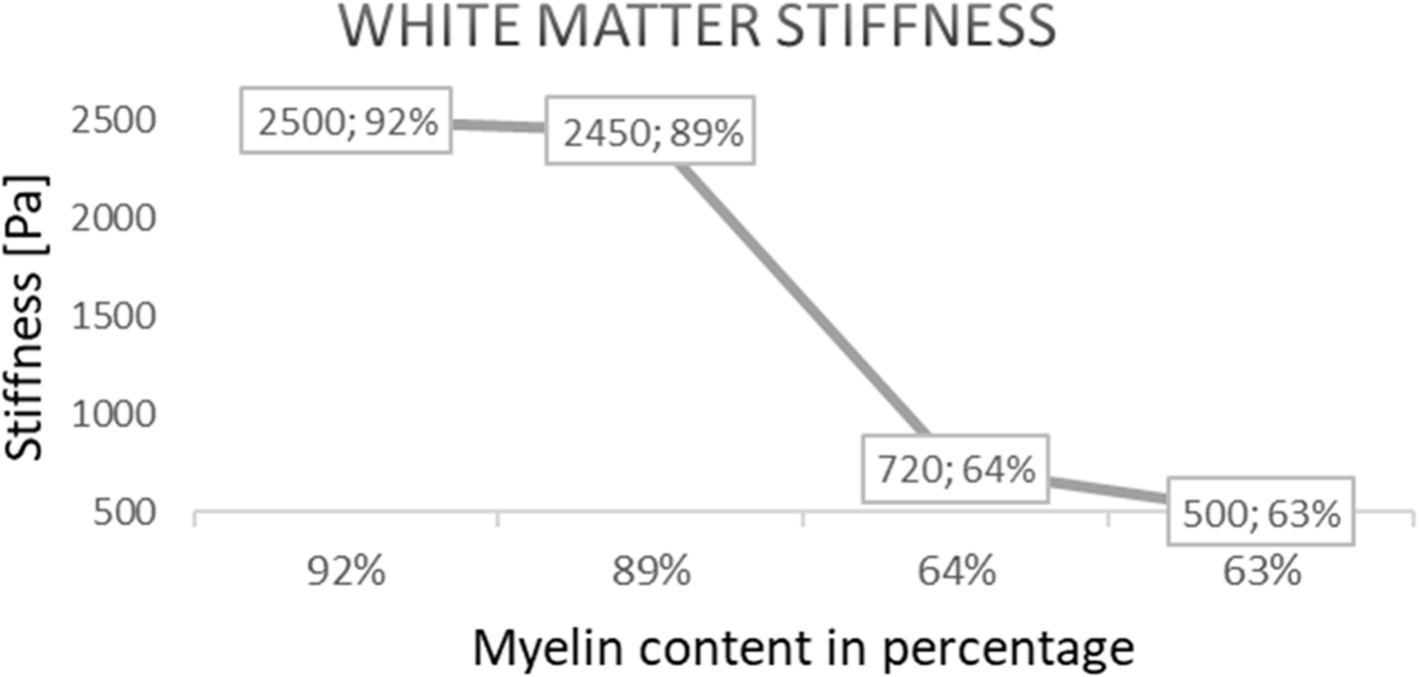
Correlation of myelin percentages with the stiffness of the white matter of a bovine brain. The plot shows the correlation of white matter stiffness in a bovine brain with myelin percentage of the tissue [38]

ECM composition is tissue-dependent and collagens, elastin, proteoglycans and glycosaminoglycans (GAGs) are some of the most characterizing fibrous proteins [9]. ECM has a dynamic structure since its organization undergoes repeated modifications in correlation with aging and pathology progression [9, 10]. The extracellular matrix guarantees the ideal environment for cell support, growth, migration, differentiation and survival [11–13]. Focusing on the brain, ECM behaves as a three-dimensional network for many physiological processes, including development regulation, tissue homeostasis and neuronal plasticity. ECM structure occupies around 20% of the brain adult volume [14]. The composition reported by Sethi et al. [15] and Hall et al. [9] showed high percentages of proteoglycans and hyaluronan, while a minor proportion is taken by collagens and fibronectin. Using cortex and cerebellum of 24-month-old mice compared to the 4-month-old mice, hyaluronic acid concentration was altered in aging and neurodegenerative diseases [9, 10, 16]. Furthermore, some proteoglycans such as decorin, chondrotin sulfate and heparin sulfate proteoglycans have some effects on neurofibrillary tangles formation and beta amyloid interactions, the two primary elements characterizing Alzheimer’s disease [17–21]. Changes in ECM composition lead to a modification of the mechanical properties (i.e. shear elasticity (μ)) and may be correlated to aging and AD onset. Nowadays the literature on the AD-related ECM features is still dependent on the techniques used. Hiscox et al. [22] used magnetic resonance elastography (MRE) to study the ECM stiffness of 12 healthy young subjects with an age between 19 and 30 (mean age 25.2 ± 3.0 years), gaining a shear stiffness physiological value in the hippocampal region of 2.89 ± 0.32 kPa. According to MRE technique, the ECM softens during aging, resulting in an annual stiffness decrease rate of ∼0.8% (0.015 kPa per year) (Table 1) [22–28]. MRE is a non-invasive technique, which combines traditional magnetic resonance imaging with acoustic waves, allowing to evaluate viscoelastic properties of soft tissues [29]. However, its resolution is affected by the long time for acquisition (minutes), enlarging the risk of possible patients’ head movements [30]. In line with this, Kalra et al. [28] combined MRE with diffusion tensor imaging (DTI) to deepen the local and directional dependency of brain tissue stiffness and confirmed the stiffness decrement. An anisotropic approach in the brain stiffness study was applied to find more details about shear stress vector orientation on different planes in space. This study reported a decrease in ECM stiffness, in accordance with the results obtained applying only MRE. In contrast to MRE and DTI results, atomic force microscopy (AFM) and indentation techniques showed stiffness increment of ∼20–150% with aging [31, 32]. Unlike MRE and DTI, AFM is an invasive imaging technique that requires the extraction of the tissue to be tested. Shear elasticity is evaluated using Van der Waals interactions forces between the cantilever tip and the tissue, resulting in the deviation of a laser light pointing to the cantilever. Thanks to the laser light reflection, the machinery is able to quantify the height of the cantilever, obtaining the sample rigidity [33, 34]. Similarly, indentation is an invasive procedure consisting in the measurement of the machine tip penetration area on the sample surface. Qian et al. [35] raised some questions about the homogeneity of the results using indentation methods. In case of brain-like soft biomaterials, indentation methods show values with high deviations due to reasons related to structural architecture and heterogeneity of the tissue: (1) the heterogeneity of the biomaterial could lead to difficulties in the test operation. Indeed, a non-flat tissue, which presents numerous asperities (e.g. the brain), shows inaccurate values; (2) in some tissues, the hypothesis of isotropy used in the analytical models for data analysis could be not accurate and this may have a repercussion on the quality of the experiments outputs; (3) a universal protocol for indentation techniques is lacking, allowing user-related variation of the boundary conditions in the experimental setup; (4) the brain stiffness changes according to the tested regions [36]. In vitro AFM and indentation present a technical limitation regarding the small size of the samples that could lead to an erroneous global stiffness measurement [34]. Moreover, it is relevant to highlight that ECM stiffness increase data were obtained only in experiments performed on mice brain samples. This aspect combined with additional data obtained from mouse and bovine models supported the ECM stiffness decrease with aging, showing a direct correlation between myelin concentration and cerebral elasticity [37, 38]. In particular, Weickenmeier et al. [38] found that in bovine brain white matter an increased percentage of myelin leads to a more stiffen tissue. Indeed, a myelin content of 63% showed a stiffness of 0.5 kPa, while a myelin content of 92% matched with a stiffness of 2.5 kPa (Fig. 3). The same Authors confirmed the correlation between myelin content and stiffness in a following study on human brain [39]. Since it has been reported a reduction in the myelin amount during aging [40, 41], it is reasonable to suppose that stiffness decreases with aging. In literature, there is a consensus about the decrement of AD patients’ ECM stiffness compared with the age-matched healthy patients (Table 2). Further experiments on this topic have been conducted on both mice and human brain tissue using different techniques. MRE and multifrequency magnetic resonance elastography (MMRE) have been performed on living subjects [42–48], while nanoindentation and AFM have been executed in vitro [49, 50]. As an example of stiffness value, in post-mortem human brain tissue has been reported a decrement in stiffness of ∼23.5% for grey matter and ∼27.9% for white matter [49]. These data suggest that ECM stiffness is region-dependent, as this has been further confirmed by experiments on mice brain and on post-mortem human brain samples [36, 51]. Results obtained using in vitro techniques (i.e. nanoindentation and AFM) on the AD hippocampal region were in accordance with the data obtained with non-invasive procedures (i.e. MRE and MMRE). As already discussed, stiffness decrease may be caused by myelin loss occurring both in aging and in AD progression [37, 38, 40, 41, 50]. In line with this, experimental observations in AD exhibited further myelin loss compared to physiological aging [40, 50].

**Table 1:** Brain ECM stiffness in aging

Method	Stiffness in aging	Samples	μ	REF
MRE	↓	24 healthy human volunteers (22–72 years old)	Hippocampal region: ◾ young: 2.89 ± 0.32 kPa ◾ older: 2.65 ± 0.39 kPa (difference: -8.30%)	[22]
MRE	↓	55 healthy human volunteers (18–88 years old)	-0.015 kPa/year in healthy brain (0.8%, p < 0.001)	[23]
MRE	↓	66 healthy human volunteers (18–72 years old)	-0.75%/ year (p < 0.001)	[24]
MRE	↓	45 healthy human volunteers (56–89 years old)	-0.011 ± 0.002 kPa/year	[25]
MRE	↓	50 healthy human volunteers (20–69 years old)	-0.0065 ± 0.0013 kPa/year in temporal lobes (p < 0.0001)	[26]
MRE	↓	54 healthy human volunteers (36–72 years old)	-0.011 kPa/year in hippocampal region	[27]
MRE + DTI	↓	28 healthy human volunteers (18–62 years old)	Both isotropic and anisotropic stiffness decrease with age in different brain regions	[28]
AFM	↑	79 C57BL/6 mice (considered from post-natal day one to 10-month-old)	Cortex region: ◾ 1-day-old: 0.255 ± 0.014 kPa ◾ 31-day-old: 0.541 ± 0.035 kPa % difference: + 112.16%	[31]
Indentation	↑	Two age groups of wild-type mice (C57BL6/ Harlan): 8 juveniles (1-month-old) and 5 adults (6 and 9-month-old)	An increase of 20%-150% with aging in hippocampal regions	[32]

Data obtained from experiments conducted on human or animal samples. Young subjects’ samples were compared to old healthy ones. For each study, the applied method (magnetic resonance elastography, atomic force microscopy or indentation), extracellular matrix stiffness variation (↑ for an increase and ↓ for a decrement), analyzed samples, shear elasticity (μ) variations and the article reference are reported

**Table 2:** Brain ECM stiffness in Alzheimer’s disease subjects in comparison with healthy condition

Method	Stiffness in AD	Samples (healthy vs. AD subjects)	μ	REF
MRE	↓	8 wild-type mice (17.5–23-month-old) and 5 transgenic APP-PS1 (AD) mice (20.5-month-old)	◾ Wild-type mice: 25.0 ± 6.4 kPa ◾ AD mice: 19.3 ± 3.3 kPa % difference: -22.80% (p = 0.0031)	[42]
MRE	↓	39 wild-type mice (6-week-old) and 45 transgenic APP23 (AD) mice (6-week-old)	Hippocampal region: ◾ Controls mice: 7.75 ± 0.3 kPa ◾ APP23 mice: 7.01 ± 0.52 kPa % difference: -9.55%	[43]
MRE	↓	28 human patients: ◾ 7 with probable AD ◾ 14 PIB-negative cognitively normal controls (CN-) ◾ 7 PIB-positive cognitively normal controls (CN +)	◾ CN- group: 2.37 kPa ◾ CN + group: 2.32 kPa ◾ AD group: 2.20 kPa (*p* = 0.0055) % difference: -7.17% (AD and CN-) and -5.17% (AD and CN +)	[44]
MRE	↓	48 human patients: ◾ 16 amyloid-negative cognitively normal controls (CN-) ◾ 16 amyloid-positive cognitively normal controls (CN +) ◾ 8 amyloid-positive subjects with mild cognitive impairment ◾ 8 amyloid-positive subjects with probable AD	◾ CN group: 2.51 ± 0.09 kPa ◾ AD group: 2.40 ± 0.09 kPa % difference: -4.38%	[45]
MRE	↓	84 human patients: ◾ 20 normal pressure hydrocephalus patients (60–86 years old) ◾ 8 AD patients (78–87 years old) ◾ dementia with Lewy bodies patients (63–76 years old) ◾ frontotemporal dementia patients (54–65 years old) ◾ 46 cognitively normal controls (56–89 years old)	A mean of -0.009 ± 0.001 kPa/year	[46]
MRE	↓	23 human patients: 11 AD patients (mean age: 76.8) and 12 healthy controls (mean age: 69.4)	Cerebrum: ◾ healthy controls: 2.50 ± 0.05 kPa ◾ AD patients: 2.25 ± 0.05 kPa % difference: -10% (*p* = 0.004)	[47]
MRE	↓	42 human patients: 21 AD patients and 21 healthy controls (mean age 75 years)	Hippocampus: ■ healthy controls: 1.076 ± 0.190 kPa ■ AD patients: 0.863 ± 0.147 kPa % difference: -19.80% (*p* < 0.001)	[48]
Indentation	↓	Post-mortem brain tissue from frontal lobes of 10 subjects: ◾ 5 AD patients ◾ 5 normal controls	-23.5% (gray matter) and -27.9% (white matter) (p < 0.0001)	[49]
AFM	↓	16 transgenic B6C3-Tg (AD) mice and 27 wild-type littermates	◾ Wild-type littermates: 0.651 ± 0.138 kPa ◾ AD mice: 0.402 ± 0.097 kPa % difference: -38.25%	[50]

ECM data comparison between healthy and pathological human or animal samples with a similar range of age. The applied method (MRE, AFM or indentation), ECM stiffness variation (↑ for an increase and ↓ for a decrement), analyzed samples, shear elasticity (μ) variations, year of the experiment conduction and the article reference are reported. Shear elasticity (μ) is a measure of the elastic shear stiffness of a material. PIB stands for “Pittsburgh Compound B”

### Nuclear lamina and AD

The nuclear lamina is a nuclear structure that represents the final component of the force transmission pathway from the extracellular matrix to the nucleus. Indeed, the nuclear lamina consists of a nuclear structure that is sensitive to extracellular matrix changes, provides support and a stress-related shield for the inner nuclear membrane. It is a meshwork composed by four intermediate filament proteins (A, B1, B2, C). Nuclear lamina is localized in the proximity of the nuclear inner membrane and it is connected to peripheral chromatin [52]. It is involved in several cell mechanisms and functions such as DNA replication, nuclear and chromatin organization, cell development and differentiation [52]. In physiological conditions, lamin A/C is highly expressed in stiff tissues, whereas it is almost absent in soft tissues such as the brain [53]. Lamin A/C enrichment, which leads to higher nuclear stiffness, may act as a genome protective agent [53, 54]. As for lamin B1, it is necessary for the nuclear shape maintenance, while lamin B2 is important for the neuronal migration during development [55, 56]. In physiological conditions, nuclear lamina is highly dynamic and sensitive to extracellular matrix variations through the mechanotransduction pathways. Indeed, like the ECM, the nuclear lamina showed significant alterations in terms of quantity and thickness during AD progression. In fact, in AD lamin A/C levels increased causing the nuclear envelope stiffening and altering the spatial arrangement of the nuclear scaffold [57, 58]. On the other hand, the lamin B1 reduction leads to a functional and morphological cell nucleus alteration [4, 57, 59]. These data were collected using ex-vivo mice or human brain samples by different techniques, such as immunohistochemistry, immunofluorescence microscopy and Western blotting (Table 3). These immunological techniques are able to evaluate the level of lamin A, lamin B1 and lamin B2 [60–62]. It was found that the levels of lamin A and B2 in neurons of AD subjects increased [63, 64], and this variation led to nuclear envelope stiffening, while lamin B2 modifications seemed not to alter the nuclear lamina localizations [55]. In opposition, the elderly and even more AD patients presented a decrease in lamin B1 percentages, suggesting a contribution to nuclear deformation [4]. Due to the lamins changes, AD has been recently considered a laminopathy [4]. A hypothesis on the mechanism leading to the lamin A increment is about the lamin B1 reduction and has been explored in non-neuronal cell types. In order to generate an upregulation of lamin A, cells activate the LMNA gene, which, in the brain tissue, is usually maintained in its silent form inside the condensed heterochromatin [57, 65–67]. Chang et al. [68] demonstrated on breast cancer cells that a decrease in lamin B1 levels may lead to heterochromatin decondensation, causing the relocalization of LMNA gene and enabling its transcription. In support to lamins B1-A correlation, Shimi et al. [69] reported that in HeLa cells an increase in lamin A was possible only by silencing lamin B1 gene. Therefore, the Authors suggested that the nucleus could induce a decrease in LMNB1 gene expression levels in order to unfold heterochromatin and increase the expression of the LMNA gene and thus, in lamin A production. These lamins relationship may be favoured by the two lamins spatial disposition. Nmezi et al. [70] conducted a study on HeLa cells, human and mice fibroblasts highlighting the formation of different lamins microdomains. Lamin B1 meshwork was located at inner nuclear membrane periphery, laying on some lamin A districts enabling a continuous interaction with them. Meanwhile, using stochastic optical reconstruction microscopy, the same Authors found lamin A localized in the nucleoplasm inner region. Although the mechanism leading lamins regulation in neurons has still to be identified, lamin A increase and lamin B1 decrease have been recently considered crucial factors in AD onset [4, 57–59]. Indeed, lamins reorganization induces nucleocytoplasmic scaffold alterations [4, 55, 65, 71], forming blebs and invaginations on the nuclear envelope [69, 72]. In particular, Matias et al. [72] reported a correlation between lamin B1 loss and invaginations in in vitro hippocampal astrocytes cultures, while Shimi et al. [69] observed lamin A rich blebs in HeLa cells experiments. Frost et al. [4] analyzed post-mortem human AD brains and showed that 60% of analyzed samples had a threefold increase of the invagination number respect to age-matched control brains.

**Table 3:** Nuclear lamina components assessment

Result	Comparison	Method	Samples	REF
**LAMIN A**				
↑	Healthy elderly case vs healthy young case	Immunofluorescence	Embryos from NMRI mice brain tissue	[63]
↑	Healthy elderly case vs healthy young case	Immunohistochemistry	Adult Sprague–Dawley rats (8 weeks old) brain tissue	[64]
↑↑	AD case vs healthy elderly case	Immunohistochemistry Immunofluorescence	Autopsied human AD brain tissue	[57]
↑↑	AD case vs both healthy young and healthy elderly cases	Western Blot	Human hippocampal samples	[58]
**LAMIN B1**				
↓	Healthy elderly case vs healthy young case	Immunohistochemistry	Adult Sprague–Dawley rats (8 weeks old) brain tissue	[64]
↓	Healthy elderly case vs healthy young case	Immunocytochemistry	ICR mice or heterozygous GAD67-GFP knock-in mice at embryonic day 17.5 hippocampal tissue	[73]
↓	Healthy elderly case vs healthy young case	Immunocytochemistry	C57Bl/6 mice and human post-mortem brain material	[72]
↓	AD case vs healthy elderly case	Post-mortem Comparative Analysis (e.g. Western Blot)	Human brain tissue	[4]
↓	AD case vs healthy elderly case	Immunohistochemistry Western Blot 3D Confocal Microscopy	3xTg and APP/PS1 mouse models of AD and human post-mortem hippocampal tissue	[59]
**LAMIN B2**				
↑	Healthy elderly case vs healthy young case	Immunohistochemistry	Adult Sprague–Dawley rats (8 weeks old) brain tissue	[64]
↑↑	AD case vs healthy elderly case	Immunohistochemistry Immunofluorescence	Autopsied human AD brain tissue	[57]

Results of the experiments about nuclear lamina conducted on post-mortem human brain samples and mice brain tissue. The variations of lamin A, B1 and B2 expression in aging and Alzheimer’s disease are reported with their related controls. The method, the samples and the reference are reported for each study. The arrows indicate the lamins concentration variation of healthy elderly case vs healthy young case and AD case vs healthy elderly case. “↑” and “↓” are used for an increase and a decrement, respectively. “↑↑” is used for a remarkable increment in lamin signal

### Nuclear transport and its impairment in AD

The nucleo-cytoplasmic transport of molecules, such as transcription factors and mRNA, is essential for cell survival and function [65, 74–76]. The nuclear pore complex (NPC) is a protein-based structure, which connects the inner and outer nuclear membranes, playing a key role in macromolecular transport from nucleoplasm to cytoplasm and vice versa [77]. NPCs are responsible for the correct maintenance of proteostasis, a process regulating the proper transport and distribution of proteins between nucleoplasm and cytoplasm. Based on electron microscopy acquisitions, its structure appears to remind a cylindrical shape with a diameter of ∼30 nm and a length of ∼50 nm [77]. NPC function is defined by the interactions between NPC binding sites and some of its constituent proteins named nucleoporins (NUPs). The NPC role in nuclear-cytoplasmic transport is dependent to the proper NUPs positioning [77, 78]. Two types of nucleo-cytoplasmic transport can be distinguished: (1) small molecules (typically up to ∼5 nm radius) diffusion by passive transport; (2) larger cargoes (> 15 nm) facilitated transport by carrier proteins [79]. Aging and AD have been correlated with the loss or alteration of essential NUPs. For instance, NUP93 is damaged and lost in aging conditions [74], while NUP98 is mislocalized in AD and contributes to tau tangles formation [80]. Interestingly, the loss or dysregulation of essential NUPs has been associated to the reduction in the number of nuclear pore complexes with consequences on the nucleo-cytoplasmic transport [80]. Eftekharzadeh et al. [80] conducted a study on AD human hippocampal sections and a study on AD mice brain samples. Combining immunostaining and electron microscopy, they found a reduction of around 300 NPCs (about 45%) in AD cases respect to the healthy cases. Moreover, nuclear blebs and invaginations occurring in AD are known to disrupt the nucleoskeleton morphology, leading to the occlusion of the NPCs [65]. Taken together, the reduction in NPCs number and the NPCs closure interfere with the nucleo-cytoplasmic molecular transport [76]. In addition, using in situ hybridization with β-actin in the hippocampal region of AD human brain samples, it was reported that protein phosphatase 2A (PP2A) subunit mRNA and β-actin mRNA decreased, revealing that PP2A mRNA could be limited in its transport through the nuclear membrane [75, 81]. Interestingly, the downregulation of PP2A in AD neurons impairs the physiological phosphorylation process of tau, involved in tangles generation and better detailed below [82, 83].

### Tau dynamics and aggregation in relation to nuclear pore transport

**Fig. 4:**
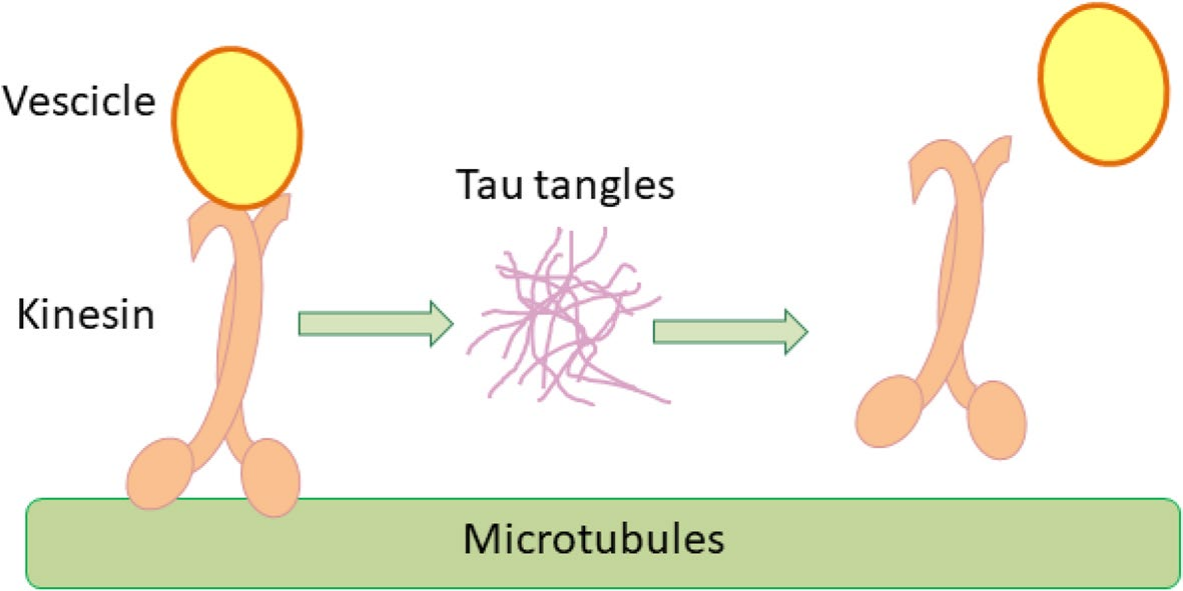
Tau tangles-driven impairment of vesicle transport on microtubules. Kinesin is the motor protein responsible for vesicles transport along the microtubules. In the presence of tau tangles, kinesin dissociates from microtubules releasing the cargo into the cytoplasm

Alternative splicing of tau leads to the production of six isoforms with a molecular weight ranging from 37 to 46 kDa and N- and C-termini are very close when tau is unbound in the cytoplasm [84]. To stabilize microtubules, tau binds the filaments with the C-terminus region, while the N-terminus remains far from both the tau C-terminus and the microtubule [84]. Through this molecular interaction, tau indirectly regulates the nucleo-cytoplasmic transport, which is strongly influenced by the microtubule stabilization [85, 86]. Indeed, microtubules act as rails for molecular transport and kinesin, which is the motor protein allowing the motion of vesicles rich of neurotransmitters throughout the cell [80, 85, 87]. As stabilizer, tau can influence microtubules dynamics, acting simultaneously with other proteins [88]. In neurons, tau is physiologically localized in the cytoplasm of the axon region [84, 89]. Hochmair et al. [90] conducted an experiment on AD human brain samples, showing tau accumulation localized throughout the whole cell. Tau was observed in cytoplasmic inclusions localized in proximity to the nucleus (∼1.5 μm distance to the nuclear envelope) and in low chromatin density areas of the nucleus, where tau had a spherical geometry. Tau was also observed close to the NPC at the nuclear side, composing a fine irregular layer of small granules in the nucleoplasm. The three different localizations were due to the alteration of tau phosphorylation level [90]. In physiological conditions, this process is balanced by activity and concentration of kinase and phosphatase. The former oversees the adding of a phosphate group to a protein, while the latter can remove a phosphate group [84, 89, 91]. PP2A and glycogen synthase kinase-3β (GSK-3β) are the enzymes responsible for tau dephosphorylation and phosphorylation, respectively [84, 89, 91]. While dephosphorylated tau strongly binds the microtubules guaranteeing their stability, phosphorylated tau shows a lower binding affinity to microtubules, resulting in its delocalization in the cytoplasm [84, 92]. During AD onset, PP2A activity decreases by almost 50%, GSK-3β increases and, thus, phospho-tau increases too [81, 83, 84, 91, 93]. Tau hyperphosphorylation was found to increase the molecular affinity to self-aggregate and to generate intracellular tangles [2, 55, 84, 94]. Studies using surface plasmon resonance measurements showed that hyp-tau interacts with the NPC, leading to NUP98 delocalization in the cytoplasm [80]. NUP98 delocalization contributes to the destabilization of the NPC arrangement and thus to the alteration of nucleo-cytoplasmic trafficking. Moreover, cytoplasmic NUP98 attracts tau molecules, promoting tau tangles agglomeration [80]. In AD, tau tangles are located in microtubules proximity and affect the kinesin-mediated transport (Fig. 4). Indeed, kinesin in proximity of tau agglomerations dissociates instantaneously from microtubules, releasing transported cargoes into the cytoplasm [2]. Stokin et al. demonstrated that βAPP, Aβ precursor, is one of the possible proteins carried by kinesin [95]. The kinesin detachment from microtubules could promote the βAPP proteolysis, leading to Aβ accumulation in the cytoplasm [96, 97]. The proteolysis process could also be influenced by the disruption of tau and βAPP interaction [98].

### Alzheimer’s disease from ECM alteration to synapse loss

**Fig. 5:**
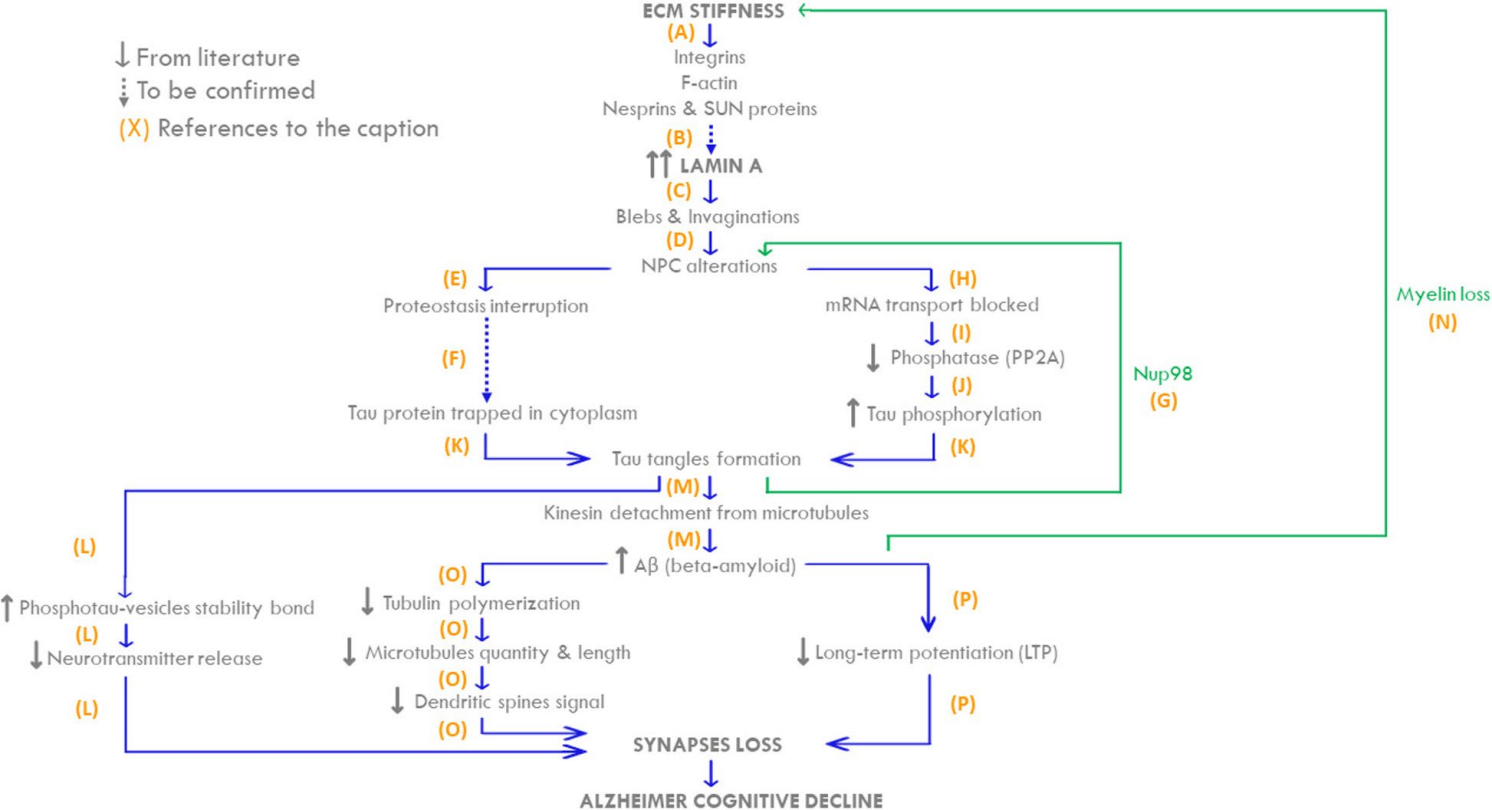
The mechanotransduction pathway from ECM to synapses failure in Alzheimer’s disease. Grey arrows “↑” and “↓” indicate increase and decrement of quantities; the continuous arrows refer to data from literature; dashed arrows stand for hypothesis; blue arrows represent the connections from literature; the green arrows are the retroaction effects. **A** ECM stiffness decreases in Alzheimer’s disease. Stimuli from ECM reach the nuclear lamina by integrins, F-actin, nesprins and SUN proteins. **B** Under mechanical stimuli, the nucleus requires an increase in lamin A quantity to protect the genome (hypothesis not yet verified). **C** The increase of lamin A exacerbates in blebs and invaginations formation, leading to the nuclear scaffold deformation and bringing to the nuclear pore complexes closure **(D)**. **E** It causes the impairment of nucleo-cytoplasmic transport of proteins, resulting in proteostasis interruption. **F** Tau nuclear-cytoplasmic transport is impaired and remains entrapped in the cytoplasmic compartment, causing a pathological accumulation (hypothesis not yet verified). **G** Hyp-tau localizes also near NPCs, releasing NUP98 in the cytoplasm and further compromising NPCs function. The release of NUP98, accelerates hyp-tau aggregation. **H** NPCs closure also causes the damage of mRNA transport, which induces PP2A gene translation impairment, a decrement of phosphatase concentration **(I)** and an increase in tau hyperphosphorylation **(J)**. **K** The high quantity of tau protein in the cytoplasm combined with tau hyperphosphorylation leads to tau self-aggregation in tangles. Tau tangles interacts stably with pre-synaptic vesicles, impeding the neurotransmitter release into the synaptic space **(L)**. **M** When tangles affect the motor protein kinesin-mediated transport, kinesin detaches from microtubules and releases the vesicles containing Aβ precursor, resulting in Aβ precursor accumulation. Aβ accumulation compromises oligodendrocytes viability, hindering their production of myelin and contributing to further ECM softening **(N).** Furthermore, Aβ accumulation reduces tubulin polymerization, leading to dendritic spines signalling loss **(O)** and alters the physiological long-term potentiation contributing to the synapses loss and progressive memory impairment **(P)**

As above stated, AD is one of the most common forms of dementia clinically characterized by memory impairment and underlining neurodegeneration with an increasing trend in prevalence [1]. Effective treatments are recently emerging [99], but they are under further development and in the meanwhile it appears relevant to continue deepening the key elements of AD aetiology and progression. Even though the brain has been considered a static organ for long time, recent studies, showing that the brain is a perfused organ, introduced the relevant role of the ECM-lamina interactions and mechanotransduction in neurodegenerative diseases (Fig. 5, A) [3, 4, 71, 100]. In the context of brain perfusion, the glymphatic system (GS) is rising interest. GS is a physiological mechanism that regulates interstitial flow throughout the cerebral parenchyma [101, 102] and it may transmit the flow-related stimuli to the neural nuclei. The aquaporin-4 (AQP4) is a key water channel in the GS, since it regulates the flow of water in and out the astrocytes that are key components of the GS. AD is characterized by the AQP4 differential expression or localization with consequent interstitial pressure increment on the softened ECM [100, 103–107]. Indeed, MRE-based studies showed the softening of ECM in aging [22–28] and a further remarkable ECM stiffness decrease in AD [42–50]. Considering the high adaptability of the nuclear lamina to external substrates [53], it is reasonable to suppose that ECM changes affect lamins variations. Indeed, lamin B1 concentration was found to decrease while, unexpectedly, lamin A levels had a notable increase [4, 57–59] (Table 4). This inverse correlation is also observed in other experiments conducted on HeLa cells and breast cancer cells [4, 57–59, 68, 69]. A possible explanation of lamin A increment in response to ECM stiffness decrease may be found by applying extrinsic forces on the HeLa cells and fibroblast cytoskeleton using magnetic tweezers [68–70]. When subjected to extrinsic forces, nuclei isolated from their ECM reorganized their lamin-based structure to modulate their stiffness. This highlights that mechanotransduction involves both the ECM and the nucleus, even if the latter keeps a plastic adaptive response when isolated [57, 106]. Even though more studies are required to verify this hypothesis (dashed arrow), the increment of pressure induced by GS combined with the decrement of ECM stiffness in AD neurons support the possibility that lamin A level increases in order to protect the neuronal genome [54, 55, 57, 106] (Fig. 5, B). Lamin variation induces nucleocytoplasmic scaffold alterations [4, 55, 65, 71], forming swellings and sinkings on the nuclear envelope called blebs and invaginations, respectively [69, 72] (Fig. 5, C). Nuclear scaffold modifications cause an impaired NPC opening due to their spatial localization on the nuclear membrane. Indeed, undergoing blebs to an imaging technique, a percentage of NPC were not visible on their surfaces [69], as declared in several works [55, 71, 74, 80] (Fig. 5, D). Because of the steric hindrance of some NPCs, some proteins remained entrapped without being able to move from the nucleus to the cytoplasm and vice versa [108] (Fig. 5, E). Indeed, protein tau exchange between nucleoplasm and cytoplasm could be altered with possible consequent cytoplasmic tau accumulation [65, 74, 76] (Fig. 5, F). Further studies will better clarify this aspect (dashed arrow). Hyp-tau also contributes to the NPC closure by promoting the dissociation of NUP98 from NPC and thus inducing their destabilization [80] (Fig. 5, G). Moreover, since blebs formation impairs NPCs, mRNA transport is also locally hindered and proteins translation is reduced [74, 75] (Fig. 5, H). For instance, in AD, this mechanism may involve PP2A protein whose expression is actually reduced [81, 93] (Fig. 5, I). The downregulation of the phosphatase PP2A levels within the AD neurons seems to be coupled with an increase in kinase quantity between the controls and the AD cases [91]. It seems that GSK-3β activity increases and phosphatase quantity decreases when tau phosphorylation is unbalanced [83, 84, 89] (Fig. 5, J). Indeed, in AD protein tau, which remains entrapped in the cytoplasm, undergoes hyperphosphorylation and self-aggregation in structures called tangles [2, 55, 82, 84, 94, 109, 110] (Fig. 5, K). Hyp-tau affects synaptic signalling by impairing neurotransmitter release from the axonal terminal. In the physiological condition, the vesicles containing the neurotransmitters migrate to the pre-synaptic terminal and they fuse with the pre-synaptic membrane. Then the neurotransmitters are released in the synaptic space by exocytosis [111, 112]. In AD, since hyp-tau loses its affinity with microtubules, it partially relocalizes in pre-synaptic terminal proximity [92]. At this point, hyp-tau interacts stably with synaptic vesicles impairing their fusion with the plasma membrane and thus the release of the neurotransmitters [111, 112]. Furthermore, a work by Jiwon Choi et al. [113] on the development of synapses suggested that in AD tau tangles impair neurotransmission not only by affecting neurotransmitter release but also contributing to synapses loss (Fig. 5, L). Meanwhile, cytosolic tangles positioned in proximity of microtubules obstacle kinesin-mediated transport, exacerbating the βAPP intraneuronal release (Fig. 4). βAPP scission generates Aβ40 and Aβ42 peptides [2, 87, 95]. Aβ oligomers in the extracellular environment tend to self-aggregate in extracellular structures named senile plaques or amyloid plaques [114] (Fig. 5, M). It is of particular interest to notice that pathological Aβ42 quantity increment compromises the viability of oligodendrocytes, which are glial cells responsible for myelin production [115, 116]. In line with these data, in AD patients it has been observed a further reduction in myelin levels in comparison with healthy elderly subjects [40, 50]. As previously highlighted, the reduction in myelin levels support the data about the ECM stiffness decrement in AD [37, 38] (Fig. 5, N). AD-related intracellular and extracellular Aβ accumulation has two main effects: α-tubulin polymerization reduction and long-term potentiation decrease. The former leads to microtubules decrement in quantity and length, as α-tubulin is their fundamental component [117]. In fact, in physiological conditions, microtubule positive terminal undergoes repetitive polymerization and depolymerization, characterizing the mature neurons dendritic spines [117–119] Changes in cell microenvironment, such as the decrease of α-tubulin polymerization could lead to axonal stretching and thus neurotransmitter axonal transport and neuronal function impairment [118, 120, 121]. Accordingly, dynamic microtubules quantity and length reduction prevent them from polymerizing along the dendrites, obstructing the proper synaptic signal transmission [117] (Fig. 5, O). On the other hand, the increment of Aβ leads to a reduction of long-term potentiation, a continuous excitatory impulse that fortifies synaptic connections [2, 122]. The impairment of long-term potentiation alters hippocampal neurons activity, leading to synapses loss and progressive memory impairment [123] (Fig. 5, P). All combined, the reduction of the network of microtubules, the long-term potentiation and the neurotransmitter release contribute to the damage of the synaptic function and the consequent memory loss, typical features of AD.

**Table 4:**
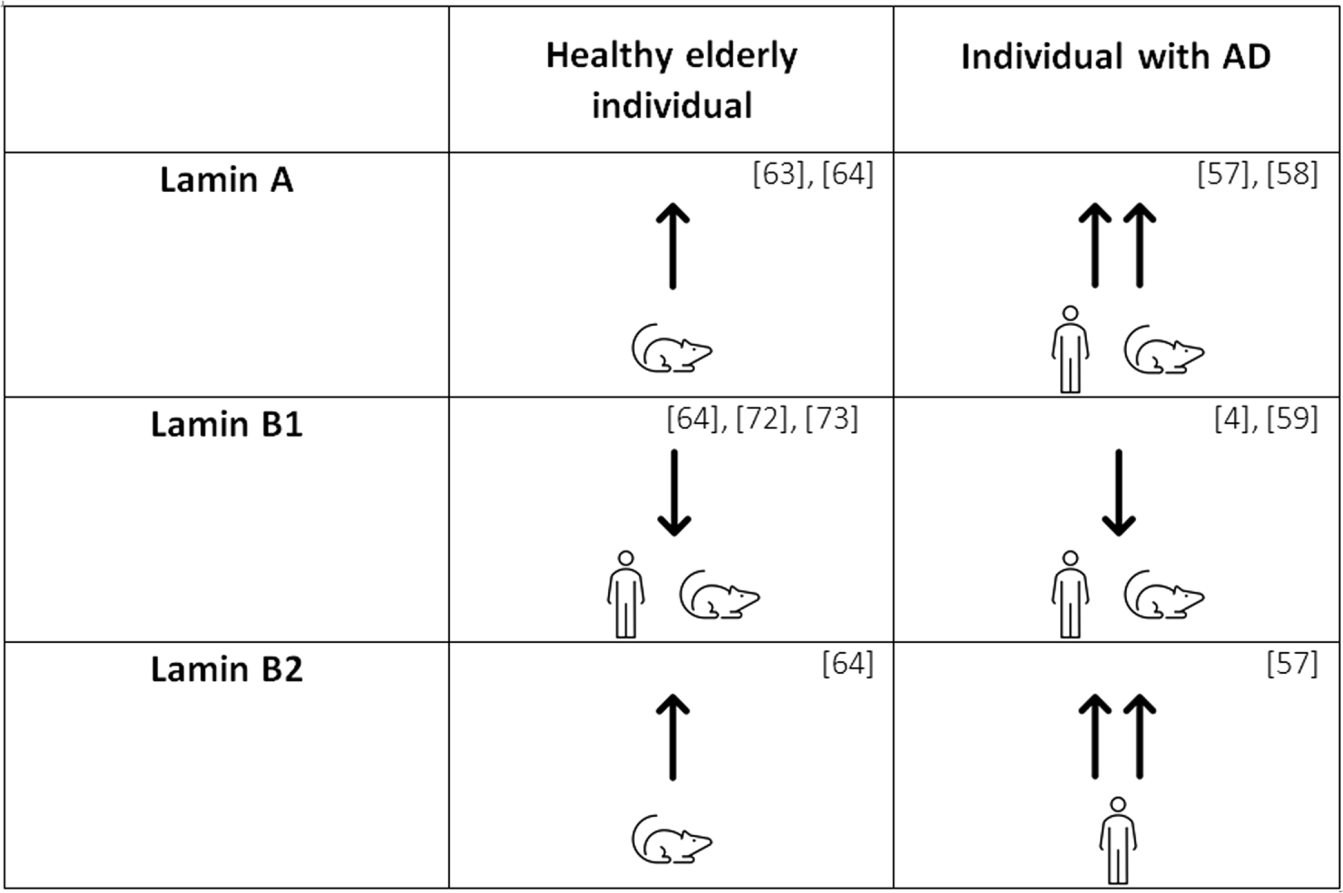
Lamins quantity changes in aging and Alzheimer’s disease respect to healthy young subjects

The experiments have been conducted on mice or post-mortem human brain samples (as shown by the sketches). “↑” and “↓” are used for an increase and a decrement, respectively. “↑↑” is used for a remarkable increment in lamins quantity

## Conclusion

In summary, this review brings evidence on how the mechanotransduction pathway from ECM to the nuclear lamina may be a player in the dynamics of AD molecular markers and vice versa (Fig. 5). In AD context, changes in physio-chemical properties of ECM affect the nuclear envelope by forming nuclear envelope-related blebs and obstructing nuclear pore complexes, leading to PP2A concentration decrease. All these dysfunctional events lead to the hyperphosphorylation of the cytosolic tau and its self-aggregation into tau tangles which impair both pre-synaptic exocytosis and microtubule-mediated transport. Therefore, the neurotransmitter release in the synaptic space and the cytosolic kinesin-mediated transport of βAPP are altered, inducing microtubules reduction in quantity and length and synaptic signal transmission impairment. Overall, the mechanotransduction pathway seems relevant in the AD context that includes the nuclear scaffold deformation and role of AD molecular markers, such as hyp-tau and Aβ. In line with this, specific aspects of mechanotransduction in neurons have been extensively discussed since 1998 (Table 5) but, to the best of our knowledge, there is no work showing the entire pathway from ECM to synapse response. To fill this gap, we have resumed all the scientific evidence supporting the whole mechanotransduction pathway from the extracellular environment to the neurons. We also focused on its implication in AD, paving the way for innovative therapeutic targets (e.g. ECM and the NL) to fight this disorder. Although most of the reported connections have already been described in the literature, the processes that induce the increase in lamin A through mechanotransduction and the correlation between proteostasis interruption and tau protein accumulation have yet to be clearly confirmed (Fig. 5, dashed arrows).

**Table 5:**
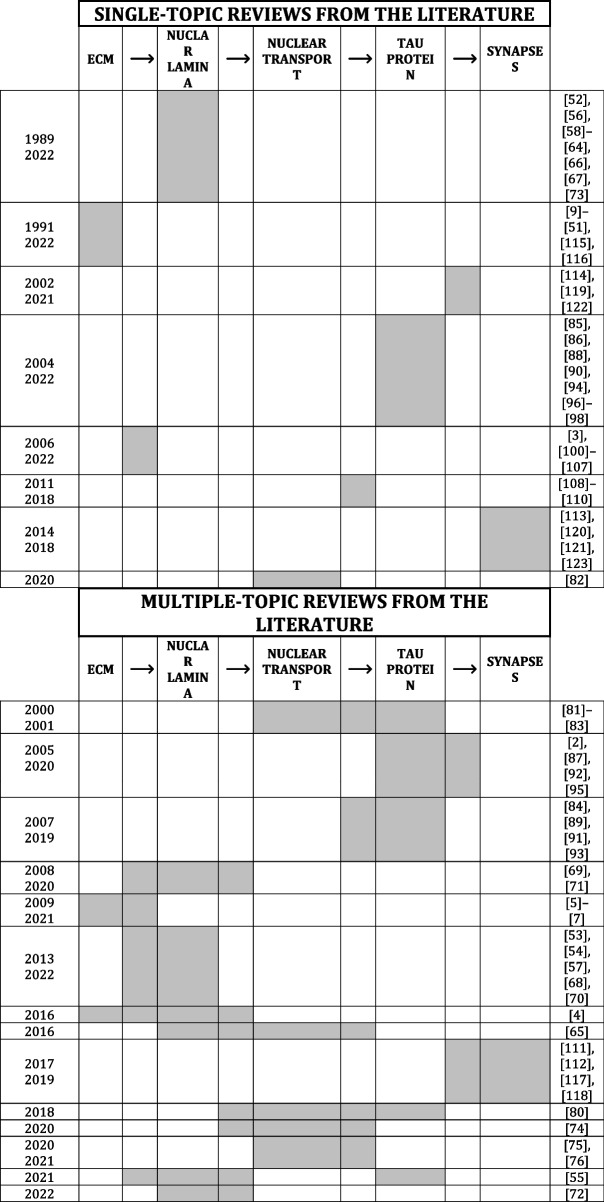
Comparison between reviews in literature and this review

Each column corresponds to a key element of mechanostransduction pathways from ECM to synapses (i.e. ECM, Nuclear lamina, nuclear transport, tau protein and synapses). Arrows (** →**) indicate the correlation between two specific mechanotransduction elements. Each row collects the review papers focused on the same topic, which is highlighted in grey. This review identified the whole pathways from the ECM to the synaptic behaviour as showed in the last row. Our review encompasses the whole pathways from the ECM to the synaptic behaviour

In conclusion, for the first time we have broadly collected current evidence correlating ECM to synapses, identifying nuclear lamina, NPCs and tau-protein as key elements in maintaining the physiological behaviour of neurons.

## Data Availability

Not applicable.

## Abbreviations

AD: Alzheimer’s disease
AFM: Atomic force microscopy
AQP4: Aquaporin-4
Aβ: Beta-amyloid
DTI: Diffusion tensor imaging
ECM: Extracellular matrix
GAGs: Glycosaminoglycans
GS: Glymphatic system
GSK-3β: Glycogen synthase kinase-3β
hyp-tau: Hyperphosphorylated tau
LINC: Linker of nucleoskeleton and cytoskeleton
MMRE: Multifrequency magnetic resonance elastography
MRE: Magnetic resonance elastography
NPC: Nuclear pore complex
NUP: Nucleoporin
PP2A: Protein phosphatase 2A
